# Transcriptomic Profiling of Hypoxia-Adaptive Responses in Tibetan Goat Fibroblasts

**DOI:** 10.3390/ani15101407

**Published:** 2025-05-13

**Authors:** Lin Tang, Li Zhu, Zhuzha Basang, Yunong Zhao, Shanshan Li, Xiaoyan Kong, Xiao Gou

**Affiliations:** 1 Faculty of Animal Science and Technology, Yunnan Agricultural University, Kunming 650201, China; zero--xc@163.com (L.T.); zhuli18328815855@163.com (L.Z.); 2 College of Animal Science, Xichang University, Xichang 615000, China; 3 Institute of Animal Husbandry and Veterinary Science, Xizang Autonomous Region Academy of Agricultural and Animal Husbandry Sciences, Lhasa 850009, China; zhazhubasang@163.com; 4 School of Animal Science and Technology, Foshan University, Foshan 528231, China; lightrainooo@163.com (Y.Z.); 15036126218@163.com (S.L.)

**Keywords:** gene–environment interactions, DEGs, machine learning, WGCNA, HIF-1 signaling pathway

## Abstract

The Tibetan goat (*Capra hircus*) exhibits remarkable adaptations to high-altitude hypoxia, yet the molecular mechanisms remain unclear. This study integrates RNA-seq, WGCNA, and machine learning to explore gene-environment interactions (G × E) in hypoxia adaptation. Fibroblasts from the Tibetan goat and Yunling goat were cultured under hypoxic (1% O_2_) and normoxic (21% O_2_) conditions, respectively. This identified 68 breed-specific (G), 100 oxygen-responsive (E), and 620 interaction-driven (I) Differentially Expressed Genes (DEGs). The notably higher number of interaction-driven DEGs compared to other effects highlights transcriptional plasticity. We defined two gene sets: Environmental Stress Genes (*n* = 632, E ∪ I) and Genetic Adaptation Genes (*n* = 659, G ∪ I). The former were significantly enriched in pathways related to oxidative stress defense and metabolic adaptation, while the latter showed prominent enrichment in pathways associated with vascular remodeling and transcriptional regulation. *CTNNB1* emerged as a key regulatory factor in both gene sets, interacting with *CASP3* and *MMP2* to form the core of the protein–protein interaction (PPI) network. Machine learning identified *MAP3K5*, *TGFBR2*, *RSPO1* and *ITGB5* as critical genes. WGCNA identified key modules in hypoxia adaptation, where *FOXO3*, *HEXIM1*, and *PPARD* promote the stabilization of *HIF-1α* and metabolic adaptation through the HIF-1 signaling pathway and glycolysis. These findings underscore the pivotal role of gene–environment interactions in hypoxic adaptation, offering novel perspectives for both livestock breeding programs and biomedical research initiatives.

## Simple Summary

The Tibetan goat (*Capra hircus*) has evolved genetic adaptations to high-altitude hypoxia. This study integrated RNA sequencing (RNA-seq), weighted gene co-expression network analysis (WGCNA), and machine learning to explore gene–environment interactions (G × E) in hypoxia adaptation. Fibroblasts derived from the Tibetan goat and lowland goat were cultured under hypoxic (1% O_2_) and normoxic (21% O_2_) conditions, revealing transcriptional plasticity as a key mechanism. Our findings provide novel insights into high-altitude livestock breeding and hypoxia-related biomedical research.

## 1. Introduction

The Tibetan goat (*Capra hircus*), native to the Himalayan mountain range, exhibits remarkable genetic and physiological adaptations to extreme high-altitude environments characterized by chronic hypoxia, low temperatures, and intense UV radiation. Key adaptations include elevated hemoglobin levels [1] and metabolic adjustments [2] that optimize oxygen utilization. However, the molecular mechanisms underlying this hypoxia adaptation remain incompletely understood.

Genomic evidence from the Tibetan goat suggests that the adaptive evolution of genes associated with cardiovascular regulation, such as *EPAS1* and *LDB2*, metabolic homeostasis maintenance, including *PAPSS2* and *DSG3*, and vascular remodeling, driven by factors like *FGF2* and *MITF*, likely forms the genetic basis of hypoxia tolerance [3,4,5]. Notably, the *PAPSS2* gene, likely introgressed from the wild goat species Capra falconeri, plays a pivotal role in reprogramming energy metabolism [3]. Selection signature analyses further revealed significant enrichment of the VEGF signaling pathway in Tibetan goat populations inhabiting Nagqu, Tibet, with core hub genes such as *EGFR*, *AKT1*, and *PTEN* promoting hypoxia adaptation by coordinating angiogenesis and cellular survival mechanisms [6]. Exome sequencing identified 339 high-altitude-selected genes, including *EPAS1*, whose Q579L missense mutation was exclusively detected in high-altitude populations [7]. At the molecular level, the HIF signaling pathway serves as a central regulatory axis, driving vascular remodeling and oxygen transport optimization through upregulation of *HIF-1α* and its downstream targets like *VEGF* [8]. These findings collectively establish a systemic framework linking genetic variations to physiological phenotypes, providing multidimensional insights into the molecular mechanisms of hypoxia adaptation in plateau-dwelling species.

High-altitude adaptation is shaped by gene–environment (G × E) interactions, yet the mechanisms governing these interactions remain elusive. Hypoxic cell culture models serve as robust tools to simulate high-altitude conditions in vitro. At the cellular level, hypoxia modulates genes in the *MMP2*, *MMP9*, and HIF-1 signaling pathways in pig cells [9,10], while yak fibroblasts exhibit distinct hypoxia-adaptive gene expression patterns under varying stress durations [11]. However, research on the Tibetan goat in this domain remains scarce. To further elucidate G × E dynamics in the Tibetan goat, we integrated transcriptomic data, weighted gene co-expression network analysis (WGCNA), and machine learning approaches to identify key genetic drivers of hypoxia adaptation. Through this study, we aim to unravel how genetic adaptations functionally interact with hypoxic stress. These findings advance novel insights into high-altitude adaptation, with implications for evolutionary biology, livestock breeding, and biomedical research.

## 2. Materials and Methods

### 2.1. Sampling of Ear Tissues from Tibetan Goat and Yunling Goat in Yunnan Province

Ear tissue samples were collected from Tibetan goats in the Diqing Tibetan Autonomous Prefecture (D, 3300 m), Yunnan Province, China, and Yunling goats in Yuanjiang (Y, 500 m), Yunnan Province, China. Five one-year-old goats were randomly selected from each group. Prior to tissue collection, ear tips were disinfected and depilated. A one-cubic-millimeter tissue fragment was aseptically excised, immediately immersed in Dulbecco’s Modified Eagle Medium (DMEM; Gibco, Miami, FL, USA) supplemented with 1% penicillin–streptomycin (P/S), and stored at 4 °C. The samples were transported to the laboratory within 12 h to maintain viability and prevent contamination.

### 2.2. Isolation, Culture and Identification of Goat Fibroblast Cells

Ear tissue samples were enzymatically digested using 0.1% collagenase type I for 4 h. Following digestion, the cell suspension was filtered and transferred into T25 culture flasks. Cells were maintained in DMEM supplemented with 10% fetal bovine serum (FBS; Gibco) and incubated at 37 °C under 5% CO_2_. Adherent cells were observed within 24 h and harvested at 90% confluency for subsequent analyses. Immunofluorescence staining was performed to confirm fibroblast identity. Fibroblasts in the logarithmic growth phase were trypsinized and seeded into six-well culture plates containing pre-sterilized glass coverslips. At 80% confluence, cells were fixed with 4% paraformaldehyde for 30 min, permeabilized with 0.5% Triton X-100, and blocked with 5% bovine serum albumin (BSA). Slides were incubated overnight at 4 °C with a primary anti-Vimentin antibody (10366-1-AP, 1:400, Proteintech, Rosemont, IL, USA) followed by a secondary Alexa Fluor 555-labeled Donkey Anti-Rabbit IgG(H + L) antibody (1:200, Beyotime, Haimen, China). Nuclei were stained with DAPI, and fluorescence images were captured using an OLYMPUS fluorescence microscope.

### 2.3. Hypoxic and Normoxic Treatment of Cell Cultures

Fibroblasts derived from Tibetan goat (D, 3300 m) and Yunling goat (Y, 500 m) ear tissue were seeded at a density of 2 × 10^5^ cells/mL in 6-well plates and pre-incubated at 37 °C under normoxia conditions (21% O_2_, 5% CO_2_) for 24 h. After pre-incubation, the cells were divided into four groups; fibroblasts from Tibetan goat were cultured under normoxic conditions (D-C, 21% O_2_, 5% CO_2_) and hypoxic conditions (D-D, 1% O_2_, 5% CO_2_), while fibroblasts from Yunling goat were cultured under normoxic conditions (Y-C, 21% O_2_, 5% CO_2_) and hypoxic conditions (Y-D, 1% O_2_, 5% CO_2_). For hypoxia treatment, cells were transferred to a tri-gas incubator (Baker Ruskinn InvivO_2_ 400, Sanford, ME, USA) and cultured under 1% O₂ and 5% CO₂ at 37 °C, balanced with N₂, for 48 h. Normoxic control cells were maintained under standard culture conditions (37 °C, 21% O_2_, 5% CO_2_) for the same duration.

### 2.4. Sample Collection, RNA Extraction and RNA Sequencing

Total RNA was extracted using TRIzol™ Reagent (Thermo Fisher Scientific, Waltham, MA, USA) following the manufacturer’s protocol. RNA concentration was measured with a Nanodrop 2000 spectrophotometer, and integrity was assessed using the RNA Nano 6000 Assay Kit (Agilent Technologies, Santa Clara, CA, USA) on an Agilent Bioanalyzer 2100 system. Only high-quality RNA samples with an RNA Integrity Number (RIN) above 7.0 were selected for subsequent library preparation. mRNA sequencing was performed on an Illumina NovaSeq 6000 platform (Illumina, Inc., San Diego, CA, USA) with paired-end sequencing (PE150, 2 × 150 bp) at Beijing Novogene (Beijing, China).

### 2.5. RNA-Seq Analysis

Raw paired-end reads were processed using Fastp (v0.12.3) [12] to remove low-quality reads (<50 bp or >6 ambiguous bases (‘N’)). Clean reads were aligned to the goat reference genome ARS1 using HISAT2 (v2.1) [13]. SAM files were converted to BAM format using Samtools (v1.13) [14]. FeatureCounts (v2.0) [15] was used to quantify raw read counts, which were normalized to Transcripts per Million (TPM). Differential expression analysis was conducted using limma (v3.46.0) [16], employing a 2 × 2 interaction model to evaluate oxygen concentration and breed factors as well as their interaction (Table 1). Differentially expressed genes (DEGs) were identified based on an adjusted *p*-value < 0.05 and |log2FoldChange| ≥ 1. DEGs were classified into three groups: breed effect DEGs (Tibetan goat vs. Yunling goat), oxygen effect DEGs (normoxia vs. hypoxia), and interaction effect DEGs (breed-specific hypoxia responses). Two custom gene sets were defined: Environmental Stress Genes (E ∪ I) and Genetic Adaptation Genes (G ∪ I). A complete list of DEGs is provided in Supplementary Table S2. Functional enrichment analysis, including Gene Ontology (GO) and Kyoto Encyclopedia of Genes and Genomes (KEGG) pathway analyses, was performed using DAVID.

### 2.6. Machine Learning Analysis

To identify key genetic factors in hypoxic adaptation, we applied Lasso regression and Random Forest approaches to environmental stress genes and genetic adaptation genes, respectively. Lasso, a machine learning technique integrating variable selection and regularization, was employed to enhance predictive accuracy [17]. The Lasso model was constructed using the “glmnet” package with L1-norm regularization, while 10-fold cross-validation ensured model robustness and minimized overfitting risks [18]. For Random Forest implementation, we ranked gene importance through mean decrease in Gini index [19], utilizing the randomForest package to build the model. Genes with importance values exceeding the 90th percentile threshold were prioritized for further analysis. To ensure biological relevance, the Boruta algorithm was concurrently implemented to validate feature importance and eliminate random noise interference. The mtry parameter was optimized through cross-validation to balance model complexity and overfitting potential. The intersection of results from both approaches served as candidate hub genes. These machine learning approaches collectively identified genetic markers potentially associated with hypoxic adaptation, providing mechanistic insights into molecular pathways underlying high-altitude adaptation.

### 2.7. Weighted Gene Co-Expression Network Analysis (WGCNA)

WGCNA [20] was conducted to identify gene modules associated with hypoxic response. The WGCNA package in R was utilized to construct a gene co-expression network, and key modules linked to hypoxia were identified for further exploration. This analysis facilitated the understanding of gene interactions and regulatory pathways involved in the cellular response to hypoxic stress.

### 2.8. Protein–Protein Interaction Network Analysis

The STRING database (http://cn.string-db.org (accessed on 15 February 2025)) was utilized to analyze the protein–protein interaction (PPI) network of both environmental stress genes and genetic adaptation genes. The resulting PPI network was visualized using Cytoscape (v3.7.1) [21]. The betweenness centrality (BC) of the network was calculated using the cytoNCA plugin, identifying the top 50 genes with the highest BC values, which would be likely to play central roles in the interaction network.

### 2.9. Quantitative RT-PCR Analysis

To validate the RNA-seq results, quantitative reverse transcription polymerase chain reaction (RT-qPCR) was performed on six selected genes, with *β-actin* used as the internal reference. cDNA was synthesized using the PrimeScript™ RT reagent Kit with gDNA Eraser (Takara, RR047A, Kyoto, Japan), following the manufacturer’s protocol. Primer sequences for each gene were designed using Primer Premier 6.25 software. SYBR Green assays were performed with TB Green^®^ Premix Ex Taq™ II (Tli RNaseH Plus) (Takara, RR820A, Kyoto, Japan) on a CFX96 Touch™ Real-Time PCR Detection System (Bio-Rad, Hercules, CA, USA). The reaction setup and thermal cycling conditions strictly followed the manufacturer’s protocols. The PCR reaction was carried out in a total volume of 25 μL containing 12.5 μL TB Green Premix Ex Taq II (Tli RNaseH Plus) (2×), 8.5 μL RNase-free water, 2 μL cDNA template, and 1 μL each of forward and reverse primers (10 μmol/mL). The amplification protocol consisted of initial denaturation at 95 °C for 30 s, followed by 40 cycles of denaturation (95 °C for 5 s) and annealing (60 °C for 30 s), with a final melt curve analysis from 65 °C to 95 °C at a ramp rate of 0.5 °C increments per step. Each sample was run in triplicate, and the relative expression levels of the genes were calculated using the 2^−ΔΔCt^ method, with *β-actin* serving as the normalization control, providing confirmation of the RNA-seq results. The primer sequences used for RT-qPCR validation are provided in Table 2.

## 3. Results

### 3.1. Cultivation and Identification of Goat Fibroblast Cells

**Figure 1:**
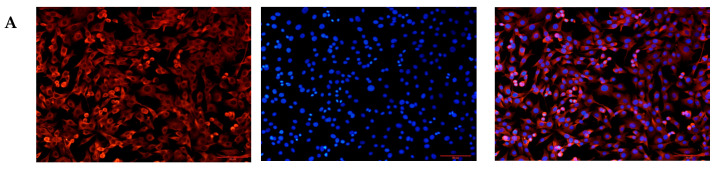

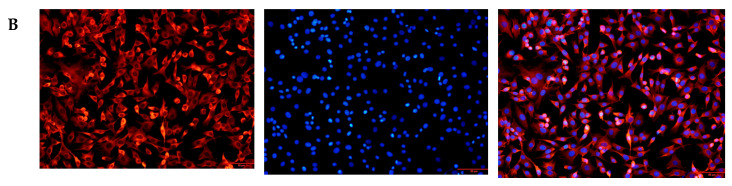
Immunofluorescence of goat fibroblast cells. (**A**) Immunofluorescence of D group goat fibroblast cells showing red fluorescence and blue cell nuclei (DAPI); (**B**) Immunofluorescence of Y group goat fibroblast cells showing red fluorescence and blue cell nuclei (DAPI).

Goat ear tissue was digested with collagenase and cultured. Cells were observed to adhere to the culture surface within 24 h. By day 4–5, spindle-shaped or fusiform cells with typical fibroblast morphology were clearly visible. After two rounds of purification using the differential adhesion method, a relatively pure population of fibroblast cells was obtained. Immunofluorescence staining was performed to confirm the identity of the isolated cells. Vimentin-positive cells exhibited red fluorescence, while the nuclei were counterstained with DAPI, displaying blue fluorescence. The merged image revealed a high percentage of positive cells (Figure 1). These results indicate that the experiment successfully isolated a highly pure population of goat fibroblast cells from ear tissue.

### 3.2. Summary of mRNA Data

**Figure 2:**
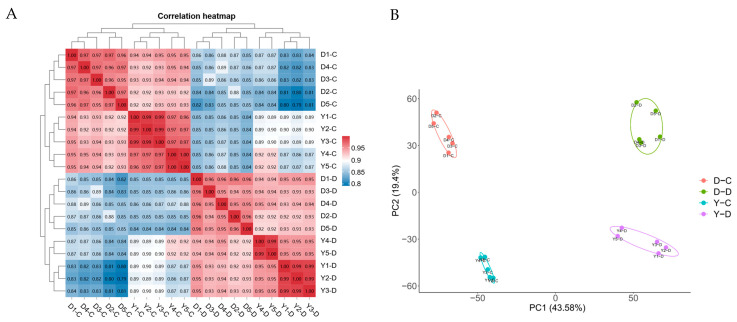
Sample structure and analysis. (**A**) Heatmap of sample correlations; (**B**) Principal component analysis (PCA) of samples.

We obtained 255.4 GB of clean bases and 1464 million clean reads from 20 samples, with an average sequencing yield of 12.77 GB per sample. The Q20 values exceeded 97%, Q30 values were above 91%, and the GC content remained stable at ~50%, ensuring high data quality (Table S1). The clean reads ranged from 63.8 M to 116.5 M, with D1-C and D4-C exhibiting the highest sequencing depth, while D5-D had the lowest. To assess data reliability, Pearson’s correlation and principal component analysis (PCA) were performed based on TPM values. Strong within-group correlations (>0.94) were observed (Figure 2A), while PCA revealed distinct clustering between groups, with PC1 explaining 43.58% of variance and PC2 19.4% (Figure 2B). These results confirm that the sequencing data were of high quality and suitable for downstream bioinformatics analyses.

### 3.3. Analysis of Differentially Expressed Genes (DEGs) and Functional Enrichment

**Figure 3:**
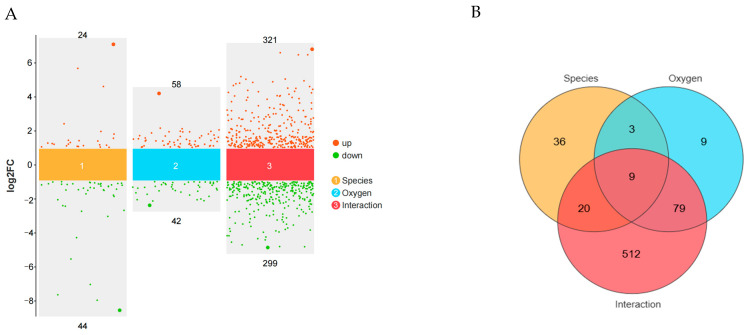
Analysis of differentially expressed genes (DEGs). (**A**) Volcano plot of DEGs with different effects; (**B**) Venn diagram of DEGs.

To identify genetic factors associated with hypoxia adaptation, differentially expressed genes (DEGs) were classified based on breed, oxygen concentration, and their interaction effects, yielding 68 DEGs in the breed effect group (24 upregulated, 44 downregulated), 100 DEGs in the oxygen effect group (58 upregulated, 42 downregulated), and 620 DEGs in the interaction effect group (321 upregulated, 299 downregulated) (Figure 3A). Further classification identified 632 environmental stress genes and 659 genetic adaptation genes based on the union of oxygen-related and breed-related DEGs, respectively (Figure 3B). A complete list of DEGs and their classifications is provided in Supplementary Table S2.

**Figure 4:**
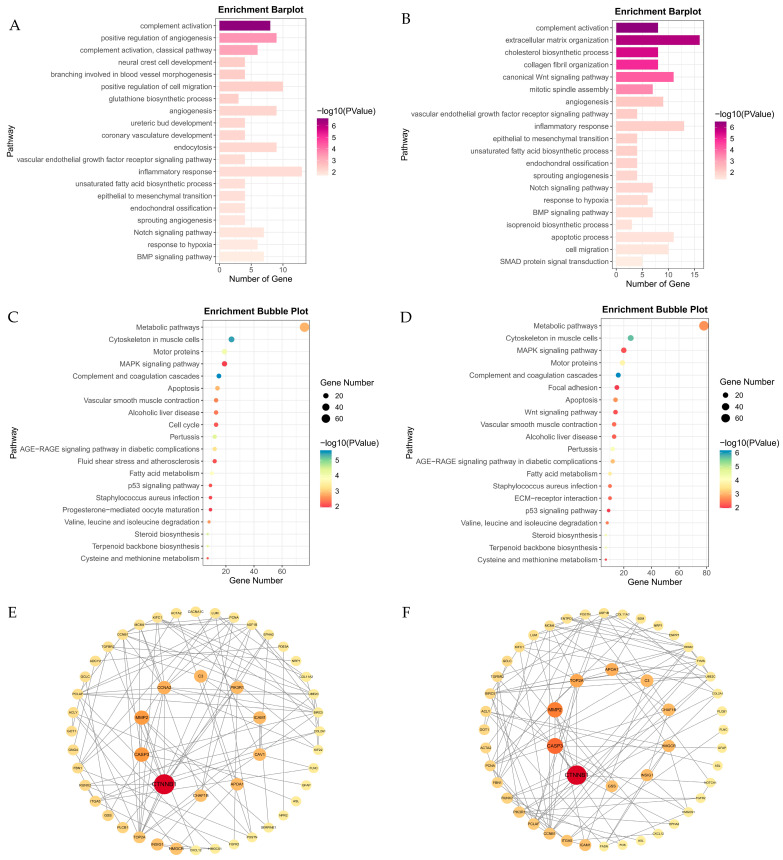
Functional enrichment and protein–protein interaction (PPI) networks. (**A**,**B**) GO biological process (BP) enrichment for environmental stress genes and genetic adaptation genes, highlighting the top 10 terms with the lowest *p*-values. (**C**,**D**) KEGG pathway enrichment, displaying the top 20 enriched pathways for each gene set. (**E**,**F**) PPI network diagrams, identifying key hub genes involved in hypoxia adaptation.

DEG analysis identified 632 environmental stress genes and 659 genetic adaptation genes based on oxygen- and breed-related effects (Figure 3A,B; Table S2). GO and KEGG analyses revealed that environmental stress genes were enriched in angiogenesis and VEGF signaling, suggesting vascular remodeling enhances oxygen delivery. Additionally, inflammatory response and glutathione biosynthesis enrichment indicate immune regulation and oxidative stress defense. KEGG pathways, including MAPK signaling, vascular smooth muscle contraction, and fluid shear stress, suggest hemodynamic adaptation, while fatty acid metabolism and apoptosis regulation highlight metabolic reprogramming (Figure 4A,C; Table S3). Genetic adaptation genes also showed enrichment in hypoxia response, angiogenesis, and blood vessel regulation, indicating long-term vascular remodeling. Enrichment of TGF-β signaling and apoptosis regulation suggests genetic modulation of cell survival. KEGG analysis further confirmed MAPK signaling, vascular contraction, and glutathione metabolism as key pathways optimizing oxygen utilization and oxidative stress resistance (Figure 4B,D; Table S4). The PPI network revealed distinct regulatory patterns between environmental stress and genetic adaptation genes. In the environmental stress network (Figure 4E; Table S5), *CTNNB1* was the central hub (BC = 27,649.34), interacting with *CASP3* and *MMP2*. Other key genes (*CAV1*, *PIK3R1*, *ICAM1*, *APOA1*) were associated with cellular response to hypoxia (GO:0001666) and cholesterol metabolism (GO:0008203), indicating that stress responses and metabolic changes play dominant regulatory roles under hypoxic stress conditions. In the genetic adaptation network (Figure 4F; Table S5), *CTNNB1* remained a central hub (BC = 24,717.29) and continued to interact with *CASP3* and *MMP2*. However, other key genes (*TGFBR2*, *RUNX2*, *FBN1*, *TOP2A*, *INSIG1*) were associated with vascular regulation (GO:0045766, GO:0001569), the BMP signaling pathway (GO:0030509), and the TGF-β signaling pathway (chx04350), suggesting that vascular and tissue remodeling, as well as transcriptional regulation, play dominant roles during long-term adaptation. Overall, the environmental stress network drives immediate responses through stress responses and metabolism, while the genetic adaptation network promotes long-term hypoxia tolerance via vascular and tissue remodeling and transcriptional regulation, highlighting the interplay between physiological stress responses and evolutionary adaptation.

### 3.4. Key Transcription Genes During Hypoxia Stress Identified by Machine Learning

**Figure 5:**
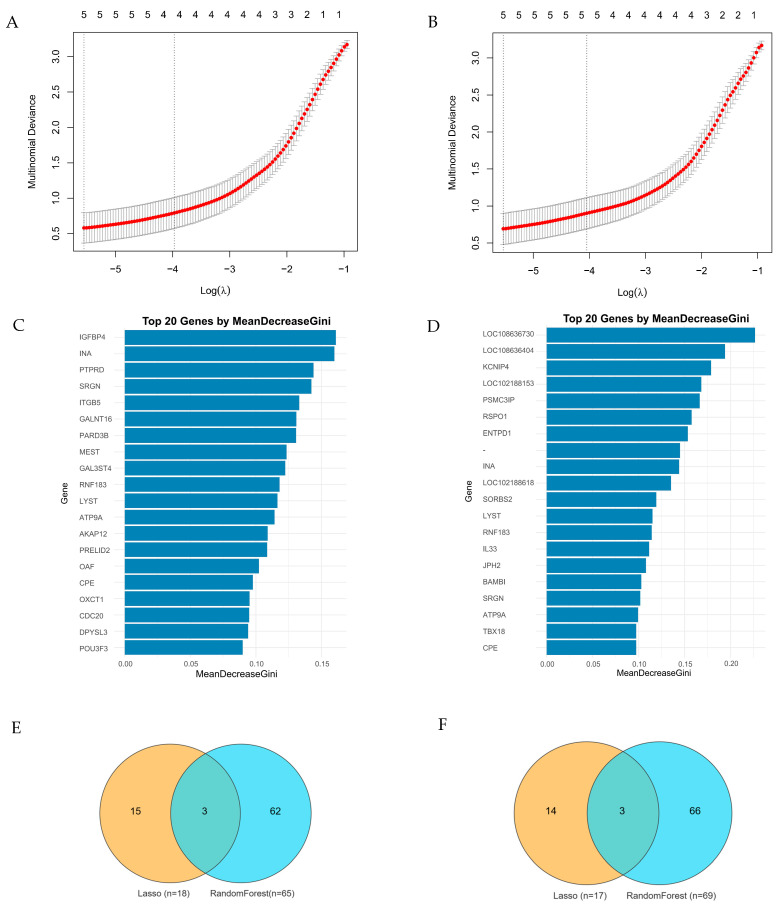
Identification of hypoxia-related genes using Lasso regression and Random Forest. (**A**) Optimization of Lasso regression parameters for hypoxia-related gene selection; (**B**) Random Forest-based ranking of key genes contributing to hypoxia adaptation, as determined by mean decrease in Gini scores. (**C**) The top 20 candidate genes identified by Random Forest in the environmental stress gene set. (**D**) The top 20 candidate genes identified by Random Forest in the genetic adaptation gene set. (**E**) The overlapping genes identified by both machine learning methods among the environmental stress genes were *MAP3K5*, *TGFBR2* and *ITGB5*. (**F**) The overlapping genes identified by both machine learning methods among the genetic adaptation genes were *RSPO1*, *TGFBR2*, and *ITGB5*.

To elucidate the genetic basis of hypoxia adaptation, we applied Lasso regression and Random Forest to identify key genes from environmental stress genes (*n* = 632) and genetic adaptation genes (*n* = 659) (Table S6). Among the environmental stress genes, Lasso regression identified 18 genes while Random Forest detected 65 genes (Table S6), with *MAP3K5*, *TGFBR2*, and *ITGB5* emerging as overlapping candidate genes between the two approaches (Figure 5E). Similarly, among the genetic adaptation genes, Lasso regression selected 17 genes whereas Random Forest recognized 69 genes (Table S6), where *RSPO1, TGFBR2*, and *ITGB5* were consistently screened by both analytical methods (Figure 5F).

### 3.5. Key Transcription Genes During Hypoxia Stress Identified by WGCNA

**Figure 6:**
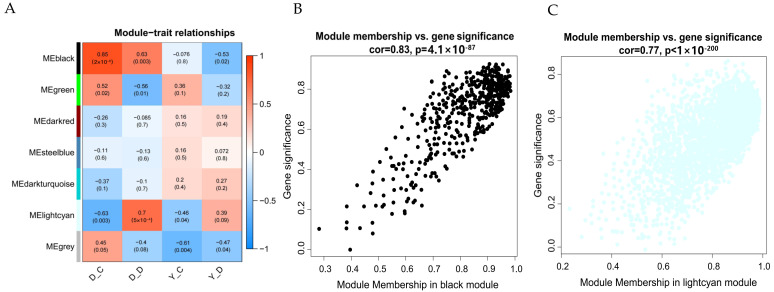

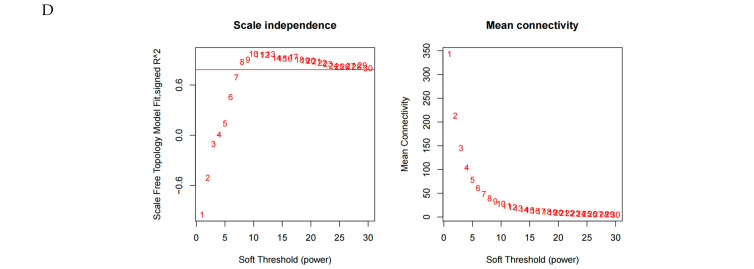
Module–trait relationships and gene significance analysis. (**A**) Heatmap showing the correlation between gene modules and traits under different conditions; (**B**) Scatter plot of MEblack module membership vs. gene significance; (**C**) Scatter plot of MElightcyan module membership vs. gene significance; (**D**) Choosing the best soft-threshold power.

The soft-threshold power in this research was calibrated to 8 (scale-free *R*^2^ = 0.85) (Figure 6D). The module-trait and gene significance analyses reveal the distinct roles of MEblack and MElightcyan in hypoxic adaptation (Table S7). The MEblack module exhibited significant positive correlations with traits in Tibetan goat cell groups (D-C: correlation coefficient = 0.85, *p* = 2 × 10^−6^; D-D: 0.63, *p* = 0.003; Figure 6A). In contrast, it showed negative correlations in Yunling goat cell groups (Y-D: correlation coefficient = −0.53, *p* = 0.02). This distinct divergence suggests that MEblack may represent fundamental adaptive mechanisms specific to high-altitude groups, potentially involving genes critical for maintaining cellular homeostasis under hypoxic conditions. Conversely, MElightcyan shows positive correlations in hypoxic conditions (D-D: cor = 0.70, *p* = 5 × 10^−4^; Y-D: cor = 0.39, *p* = 0.02) and negative correlations in normoxia (D-C: cor = −0.63, *p* = 0.003; Y-C: cor = −0.45, *p* = 0.03), highlighting its key role in hypoxic stress responses (Figure 6A). Further analysis of module membership (MM) vs. gene significance (GS) confirms these roles, with MEblack showing a strong MM–GS correlation (cor = 0.83, *p* = 4.1 × 10^−87^; Figure 6B) and MElightcyan exhibiting a robust correlation (cor = 0.77, *p* < 1 × 10^−200^; Figure 6C). These results suggest that MEblack mediates long-term hypoxia adaptation, whereas the MElightcyan module regulates hypoxic stress responses, particularly in the Tibetan goat.

To investigate the role of the black and light cyan modules in hypoxia adaptation, we integrated GO_BP, KEGG pathway enrichment, and PPI network analyses, revealing key molecular mechanisms of hypoxia tolerance in plateau animals.

**Figure 7:**
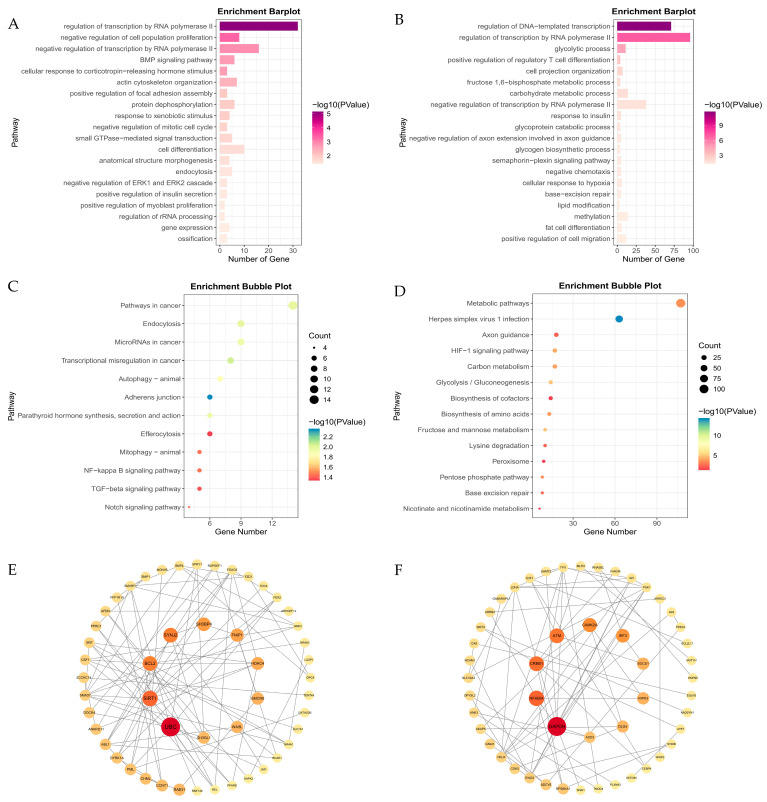
Enrichment and PPI network analysis of black and light cyan modules. (**A**,**B**) GO_BP enrichment in the black (**A**) and light cyan (**B**) modules; (**C**,**D**) KEGG pathway enrichment in the black (**C**) and light cyan (**D**) modules; (**E**,**F**) PPI networks of the black (**E**) and light cyan (**F**) modules.

In the black module (Table S8), GO_BP enrichment (Figure 7A; Table S9) identified transcriptional regulation (GO:0006357) and the BMP signaling pathway (GO:0030509). KEGG analysis (Figure 7C; Table S9) further revealed enrichment in the Notch signaling pathway (chx04330), NF-κB signaling pathway (chx04064), TGF-β signaling pathway (chx04350), and autophagy (chx04140), highlighting the role of gene transcription regulation. PPI analysis (Figure 7E; Table S11) identified the core genes *UBC*, *SMCR8*, and *WASL*, which are involved in pathways related to autophagy (chx04140, chx04137). Additionally, other key genes participate in RNA and protein synthesis processes, further reinforcing the role of the black module in transcriptional regulation during long-term hypoxia adaptation.

In the light cyan module (Table S8), GO_BP analysis (Figure 7B; Table S10) identified cellular response to hypoxia (GO:0071456) and glycolysis (GO:0006096), including key glycolytic genes such as *EGLN1*, *EGLN3*, *GPI*, *ENO1*, *PGK1*, and *PFKP* that support ATP production under hypoxia. KEGG analysis (Figure 7D; Table S10) further emphasized the HIF-1 signaling pathway (chx04066) and glycolysis/gluconeogenesis (chx00010), highlighting the roles of stress response and metabolic changes under hypoxic stress. In PPI analysis (Figure 7F; Table S11), the core gene *GAPDH* was involved in glycolysis (GO:0006096, chx00010) and the HIF-1 signaling pathway (chx04066).

These findings suggest that the light cyan module, together with the environmental stress genes, primarily responds to hypoxic stress through stress responses and glycolytic metabolism. In contrast, the black module, together with the genetic adaptation genes, mainly participates in long-term hypoxia adaptation through its role in transcriptional regulation.

### 3.6. RT-qPCR Validation of RNA-Seq Results

**Figure 8:**
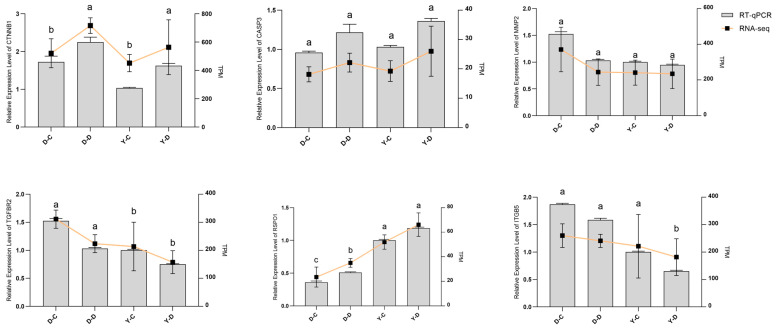
Different letters (a, b, c) indicate significant differences between groups (*p* < 0.05, Duncan’s multiple range test), while the same letter indicates no significant difference.

To validate the RNA-seq results, RT-qPCR was performed on six randomly selected genes with β-actin as the internal reference. The selected genes, including *CTNNB1*, *CASP3*, *MMP2*, *TGFBR2*, *ITGB5*, and *RSPO1*, represent a subset of candidate genes screened in this study. The findings revealed that the expression levels of these genes exhibited consistent trends with the RNA sequencing results (Figure 8). These results confirm the reliability of the RNA-seq data and highlight the functional relevance of the identified genes in Tibetan goats’ adaptation to high-altitude environments.

## 4. Discussion

Gene–environment interactions (G × E) are central to hypoxia adaptation, with transcriptional plasticity playing a more dominant role than fixed genetic variation [22,23]. Our findings demonstrate that the number of DEGs driven by interaction effects (*n* = 620) far exceeded those associated with breed effects (*n* = 68) or oxygen effects (*n* = 100), highlighting the critical role of gene–environment interactions in the Tibetan goat’s hypoxic adaptation mechanisms. Functional categorization revealed two distinct sets of genes: environmental stress genes (*n* = 632), enriched in oxidative stress response, immune regulation, and metabolic adaptation, which promote hypoxic stress responses through cytokine signaling and hemodynamic adaptation; and genetic adaptation genes (*n* = 659), associated with transcriptional regulation, TGF-β signaling, and apoptosis regulation, which support long-term structural changes to tolerate hypoxia. Further identification via machine learning found *TGFBR2* and *ITGB5*. *TGFBR2*, a receptor for TGF-β cytokine, forms a heterodimeric complex with *TGFBR1*. Its combination with TGF-β regulates many physiological and pathological processes. Studies show that in pulmonary hypertension models, *TGFBR2*’s transcriptional activation under hypoxia is suppressed, and the interaction between its promoter and distal enhancer is weakened [24]. In prostate cancer research, hypoxia-induced *EZH2* causes hypermethylation of the *TGFBR2* promoter, reducing its expression [25]. In this study, the expression of *TGFBR2* was downregulated under hypoxia, which is consistent with previous studies. As an integrin β subunit member, *ITGB5* is proven to be involved in ROS and angiogenesis regulation [26,27], and it may participate in hypoxia regulation through this pathway. However, its mechanism needs further exploration.

In the PPI networks of two different gene sets, *CTNNB1* (Catenin Beta-1) was found to be in the central position. *CTNNB1* is a key component of the canonical Wnt signaling pathway. *HIF-1α* can activate Wnt signaling by binding to its key components [28]. In fibroblasts, *CTNNB1* (β-catenin) is essential for *HIF-1α* accumulation, and its knockdown reduces *VEGF* secretion. Therefore, fibroblasts can activate *VEGFR2* through *HIF-1α* -and *CTNNB1*-dependent mechanisms [29]. *CASP3*, which interacts with *CTNNB1* in the PPI network, is targeted by miRNAs in yaks and activated in hypoxia to reduce hypoxia - induced apoptosis [30,31]. *MMP2*, which belongs to the matrix metalloproteinase (MMP) family, has a wide range of functions in hypoxic conditions. Its homeostasis is crucial for regulating hypoxia-induced pulmonary vascular remodeling [32] and also serves as a key factor in the hypoxic response of adipocytes in mice [33]. Additionally, under hypoxic conditions, the expression of *MMP2* and *MMP9* increases, accompanied by the activation of nuclear factor-κB (NF-κB)/*HIF-1α* [34].

WGCNA identified two hypoxia-related modules: the black module, predominantly active under chronic hypoxia, was enriched in transcriptional regulation and BMP signaling pathways [35,36], while the light cyan module, associated with hypoxic stress, was enriched in HIF-1 signaling and glycolysis, regulating hypoxia-induced metabolic reprogramming [37,38,39]. Transcriptional regulation represents a core mechanism of hypoxia adaptation, enabling organisms to modulate gene expression in response to oxygen deprivation [40]. In the black module, our study highlighted the enrichment of key pathways, including RNA polymerase II-mediated transcriptional regulation (GO:0006357, GO:0000122), driven by transcription factors such as *FOXO3*, *HEXIM1*, and *PPARD*. *FOXO3*, a member of the FOXO transcription factor family, plays a central role in regulating metabolism, oxidative stress responses, and cell cycle progression. Hypoxia-induced suppression of the tricarboxylic acid (TCA) cycle and reduced α-ketoglutarate levels inhibit prolyl hydroxylase domain (PHD)-mediated prolyl hydroxylation of *FOXO3*, thereby decreasing its degradation via the ubiquitin–proteasome pathway—a stabilization mechanism analogous to HIF under hypoxic conditions [41]. *HEXIM1* regulates RNA polymerase II activity by controlling the localization and activity of positive transcription elongation factor b (pTEFb) and modulates erythroid gene expression and function. Overexpression of *HEXIM1* promotes erythrocyte proliferation and fetal hemoglobin expression [42]. *PPARD* (peroxisome proliferator-activated receptor delta) protects cells under hypoxia through multiple mechanisms, such as stabilizing *HIF-1α*, promoting vascular repair [43,44], and alleviating inflammation. Hypoxia upregulates and activates *PPARD* in endothelial cells, which stabilizes *HIF-1α* protein by preventing its ubiquitin-mediated degradation [45].

Furthermore, the black module was enriched in the BMP signaling (GO:0030509) and TGF-β signaling pathways (KEGG: chx04350). Under hypoxia, the expression of *BMP2* and *BMP4* is upregulated, activating BMP signaling—consistent with our findings [46,47]. The BMP signaling pathway regulates intracellular signaling and transcriptional activation via Smad-dependent pathways. For instance, it inhibits PHD enzyme activity, prolongs the half-life of *HIF-1α*, and forms a positive feedback loop to enhance hypoxia adaptation [46,48]. Both TGF-β and BMP signaling pathways belong to the TGF-β superfamily, with hypoxia serving as an upstream trigger for TGF-β signaling. The deubiquitinase *USP9X*, a downstream effector of TGF-β, stabilizes *HIF-2α* through hydroxylation- and ubiquitination-dependent mechanisms under hypoxia, thereby promoting cellular adaptation [49]. These findings underscore the multi-layered adaptive strategies employed by the Tibetan goat to maintain oxygen homeostasis under chronic hypoxia, balancing immediate transcriptional responses with long-term structural remodeling.

In the light cyan module, we observed enrichment of cellular response to hypoxia (GO:0071456) and the HIF-1 signaling pathway (KEGG: chx04066). The HIF-1 signaling pathway is pivotal for hypoxia adaptation. Under normoxia, the *HIF-1α* subunit is hydroxylated by prolyl hydroxylases, recognized by von Hippel–Lindau (VHL) protein, and targeted for ubiquitination and degradation. Under hypoxia, hydroxylation of *HIF-1α* is inhibited, allowing its stabilization and nuclear translocation, where it dimerizes with *HIF-1β* to activate hypoxia-response elements. *EGLN1* and *EGLN3*, which directly regulate *HIF-1α* stability via oxygen-dependent hydroxylation [50,51], were identified in our analysis. This regulatory network is evolutionarily conserved in high-altitude species, including yaks [52,53], Tibetan pigs [54], Tibetan chickens [55], and Tibetan sheep [37], whereas lowland species exhibit limited hypoxia tolerance, highlighting the evolutionary significance of *HIF-1α* regulation [40]. Our findings reinforce this evolutionary convergence, emphasizing its critical role in shaping hypoxia tolerance. Under hypoxia, the expression of *NDRG1* is upregulated, aligning with our results [56]. *NDRG1* modulates the PI3K-Akt, p53, and HIF-1 signaling pathways, influencing cell growth, differentiation, and stress responses, and plays a key role in hypoxia-induced vascular remodeling [57]. *VEGFA*, a canonical *HIF-1α* target gene, is induced under hypoxia to stimulate angiogenesis, thereby enhancing oxygen and nutrient delivery to tissues—a critical mechanism for hypoxia adaptation [58]. This gene has also been observed in our study and is implicated in vascular remodeling to improve oxygen transport and blood supply in high-altitude species, including humans [59,60], yaks [61], Tibetan pigs [62], Tibetan sheep [8], and plateau zokors [63].

Additionally, the turquoise module showed enrichment in glycolysis (GO:0006096) and glycolysis/gluconeogenesis (KEGG: chx00010). Metabolic adaptation is essential for maintaining ATP production under hypoxia, and Tibetan goats exhibit a shift toward anaerobic metabolism. Key glycolytic enzymes, including *GAPDH*, *PGK1*, *PFKP*, *PDK1*, *ENO1*, *ENO2*, and *LDH-A* [64,65], were upregulated in our study, with *GAPDH* occupying a central position in the PPI network. This indicates enhanced glycolysis as a primary energy supply pathway under hypoxic stress. Similar metabolic adjustments are observed in yaks, which optimize energy metabolism to survive extreme high-altitude environments [37], and in other plateau animals such as Tibetan chickens and Tibetan sheep, where pathways related to carbohydrate metabolism are also enriched [66,67]. Beyond glycolysis, Tibetan goats demonstrate enhanced fatty acid metabolism and mitochondrial efficiency, as evidenced by upregulation of *SIRT3* and *CAMK2A* in the turquoise module [68,69]. These adaptations parallel those observed in Tibetan sheep and yaks, where fatty acid oxidation reduces reliance on oxygen-intensive ATP synthesis [70,71].

In summary, the Tibetan goat employs multi-layered genetic and molecular mechanisms to synergistically adapt to high-altitude hypoxia. Environmental stress genes mediate acute hypoxic responses through cytokine signaling and hemodynamic adaptations, while genetic adaptation genes drive long-term structural remodeling via transcriptional regulation and epigenetic modifications. The core of this adaptation lies in the dynamic integration of gene–environment interactions (G × E) with transcriptional regulatory networks and key signaling pathways, forming a plasticity-driven, multi-level adaptive system. This system balances hypoxic stress responses with chronic oxygen homeostasis, ultimately achieving synergistic coordination between hypoxia tolerance and energy metabolism efficiency.

## 5. Conclusions

The findings highlight a hypoxia adaptation mechanism in the Tibetan goat characterized by a multi-layered adaptive system dominated by transcriptional plasticity, driven by gene–environment (G × E) interactions that integrate dynamic transcriptional regulatory networks with critical signaling pathways. Future studies should integrate metabolomics and proteomics approaches to systematically analyze the metabolic profiles and protein expression patterns in high- and low-altitude Tibetan goat populations. This multi-omics framework will comprehensively dissect hypoxia-associated dynamic changes in metabolic pathways, identify key metabolite variations, and map protein functional regulatory networks. By integrating metabolomic data (including TCA cycle intermediates and lipid metabolites) with proteomic data (such as oxidative phosphorylation-related enzymes, HIF signaling pathway proteins, and their post-translational modifications), and correlating these findings with transcriptomic profiles, we aim to elucidate the synergistic mechanisms between metabolic remodeling and genetic/epigenetic regulatory factors. Such integration will enable the construction of a multidimensional “gene–protein–metabolite” interaction model to pinpoint core molecular hubs underlying high-altitude adaptation.

Furthermore, the conservation and utilization of Tibetan goats’ unique genetic resources will not only safeguard genetic diversity but also facilitate molecular marker development for breeding programs through the establishment of multi-omics databases. The hypoxia adaptation mechanisms of Tibetan goats provide a distinctive cross-species model for biomedical research. Deciphering their genetic and physiological strategies in extreme environments may yield novel insights into human hypoxia-related pathologies and therapeutic targets, particularly in advancing treatment strategies for ischemic diseases such as myocardial infarction and stroke.

## Figures and Tables

**Table 1:** Coefficients and interpretations of the 2 × 2 interaction model for differential expression analysis.

Coefficient	Comparison	Interpretation
Intercept	(D-D + D-C + Y-D + Y-C)/4	Grand mean
Strain1	(D-D + D-C − Y-D − Y-C)/4	Species effect (Breed effect)
Treatment1	(D-D + D-C + Y-D + Y-C)/4	Environment effect (Oxygen effect)
Strain1:Treatment1	(D-D − D-C − Y-D + Y-C)/4	Interaction effect

**Table 2:** Primer sequences used for RT-qPCR validation for RNA-seq results.

Gene	Forward Primer (5’ → 3’)	Reverse Primer (5’ → 3’)
*CTNNB1*	GGTGTGGGTAATAGAAC	AGAAAAACAGAAAAGGT
*CASP3*	CACCTCTAAATCTAACC	AGTCTCAACTACCCAAC
*MMP2*	ATGCCATCCCTGATAACC	CTTCCGAACTTCACGCTC
*TGFBR2*	GTTTGTTTTCTCCTTAT	ATTCACATTCCTATTTT
*ITGB5*	CCACCTGCTGCCTCTCA	ACTCGTTGGCCTCGTTC
*RSPO1*	CACACCCTCTCTGTCCCC	GCCTCTGTCTCTTCCCCT
*β*-*actin*	AGATGTGGATCAGCAAGCAG	CCAATCTCATCTCGTTTTCTG

## Data Availability

The data presented in this study are available on request from the corresponding author.

## Supplementary Materials

The following supporting information can be downloaded at: https://www.mdpi.com/article/10.3390/ani15101407/s1. Table S1: Statistical analysis for RNA-seq; Table S2: Summary of differential expression genes; Table S3: Enrichment analysis of environmental stress genes; Table S4: Enrichment analysis of genetic adaptation genes; Table S5: Key PPI genes identified in environmental stress and genetic adaptation networks; Table S6: Potential hypoxia adaptation genes identified by Lasso and Random Forest; Table S7: Genes of each module by WGCNA (0.7); Table S8: Key genes in the black and light cyan modules with module membership and gene significance; Table S9: Enrichment analysis of WGCNA (black); Table S10: Enrichment analysis of WGCNA (light cyan); Table S11: Key PPI genes with high betweenness in the black and light cyan modules.
